# Ileal intussusception secondary to both lipoma and angiolipoma: a case report

**DOI:** 10.4076/1757-1626-2-7099

**Published:** 2009-07-30

**Authors:** Ali Aminian, Morteza Noaparast, Rasoul Mirsharifi, Mohammad Bodaghabadi, Omid Mardany, Fouzeyah A H Ali, Faramarz Karimian, Karamollah Toolabi

**Affiliations:** 1 Department of General Surgery, Imam Khomeini Hospital Complex, Tehran University of Medical SciencesTehranIran; 2 Department of Pathology, Imam Khomeini Hospital Complex, Tehran University of Medical SciencesTehranIran; 3 Department of Radiology, Mubarak Al-Kabeer Hospital, Kuwait UniversityJabriyaKuwait

## Abstract

Lipoma and angiolipoma are common benign neoplasms that occur in the subcutaneous tissue and rarely in the gastrointestinal tract. These tumors are usually asymptomatic but may present with abdominal pain, bleeding and obstruction. We present a 53-years-old woman with abdominal discomfort for several weeks accompanied with bloody diarrhea and recurrent vomiting. Ileo-ileal invagination was diagnosed by computed tomography scan. Laparotomy revealed five intraluminal masses that caused intussusception. Histopathological study showed that one was angiolipoma and other lesions were lipoma. We have described some aspects of diagnosis and treatment of this rare cause of intestinal intussusception.

## Introduction

Lipoma and angiolipoma may develop as a benign tumor in all organs and rarely in large or small intestine. The majority of lipomas in the small bowel are solitary. Approximately 5% are multiple [1]. Angiolipoma constitutes histologically of adipose tissue by a vascular component that commonly located in the subcutaneous tissue of trunk and extremities. In the gastrointestinal tract, they may protrude as an intraluminal submucosal mass, which may lead to severe symptoms [2]. We describe this rare case and review some aspects of diagnosis and treatment of intestinal intussusception.

## Case presentation

**Figure 1.**
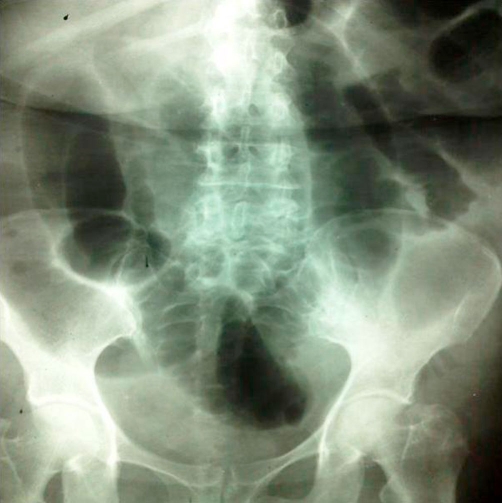
Abdominal X-Ray in favor of bowel obstruction.

**Figure 2.**
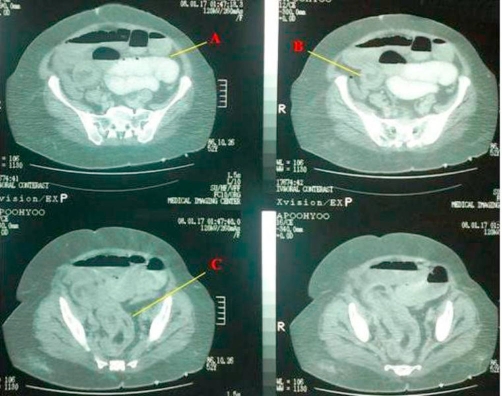
Computed tomography of abdomen showing **(A)** distention of small intestine and air-fluid level, **(B)** target lesion that characteristic for intussusceptions, **(C)** air between intussuscepted two lumens of ileum.

**Figure 3.**
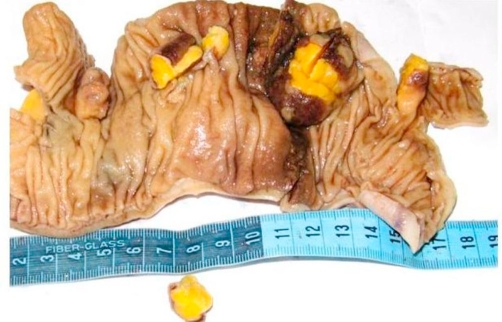
Specimen of resected Ileum revealing multiple small lesions as a lead point of intussusception.

**Figure 4.**
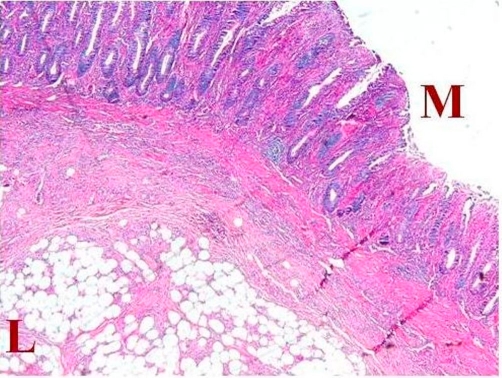
Low power pathologic appearance showing lipomatous **(L)** neoplasm adjacent to the mucosal layer **(M)**.

A 53-years-old Iranian woman was admitted to the emergency department in a tertiary referral hospital with 4 months history of intermittent upper abdominal pain accompanied with nausea and bloody diarrhea. She also had history of obstipation and constipation. These symptoms were aggravated in the recent two weeks. In past medical history, there was only a history of hysterectomy 11 years ago. There was no familial history of any disease, with no significant habitual history. In physical examination, the abdomen was distended and tender. Results of routine laboratory examinations were within normal limit. Abdominal X-ray showed dilated loops of small intestine which was indicated an obstructive pattern (Figure 1). After resuscitation, computed tomography (CT) scan was done which was showed dilatation of small intestine because of ileo-ileal invagination (Figure 2). On exploratory laparotomy, an ileo-ileal intussusception was found. After manual reduction, five intraluminal masses were palpated. Segmental ileal resection and primary anastomosis were performed (Figure 3). Postoperative course was uneventful and she was discharged with good condition after 7 days of surgery. On gross and histopathologic examination of resecetd small bowel, multiple well delineated lipomatous neoplasms one with features of angiolipoma were detected (Figure 4).

## Discussion

Intussusception occurs when a segment of intestine invaginates into itself. Intussusception accounts for about two percent of bowel obstructions in adults. In adults, intussusception is more likely to present insidiously with vague abdominal symptoms and rarely presents with the classic triad of vomiting, abdominal pain and passage of blood per rectum, making diagnosis difficult. Intussusception can be confidently diagnosed on CT scan because of its virtually pathognomonic appearance. On CT scan, intussusception appears as a “sausage-shaped” mass in the longitudinal axis, and as a “target” mass in the transverse axis. In contrast to children, more than 90% of intussusceptions in adults have a demonstrable cause, 60% due to neoplasm (60% malignant, 40% benign). The high rate of pathologic lead points, along with the high rate of malignancy, makes surgery mandatory in these cases [3].

The lipomas are rare benign tumors, representing 2.6% of non malignant tumors of the intestinal tract [4]. Most occur in colon which constitutes 65% to 75% of cases in comparison with small intestine which constitutes 20% to 25% [5]. Lipomas in the small intestine occur mainly in elderly patients and they are often single submucosal intraluminal masses. It lends an intense yellow color to the mucosa because of the underlying accumulated fat [6]. The symptoms were found in less than one-half of the patients, usually because of intussusception, obstruction or hemorrhage. Severe bleeding may occur in the form of hematemesis or melena. This is caused by superficial ulceration of the overlying mucosa [1]. A submucosal lipoma can be diagnosed if a smooth well-circumscribed mass of fat density (-50 to -100 Hounsfield Units) is revealed within the lumen of the bowel or intussuscipiens. Surgery becomes necessary if lipoma is symptomatic (obstruction and bleeding), if it is larger than 25 mm, or if the lesion mimics malignancy [7].

Angiolipoma was demonstrated by Bowen in 1912 for the first time. Howard and Helwig described its clinicopathological characteristics in 1960. Since then, angiolipoma has been regarded as a new entity [8]. Angiolipomas usually develop as encapsulated subcutaneous tumors; commonly occurring in young adults and usually located in multiple places in arms and trunk. It is usually painful and histologically it is comprised of mature adipose tissue with vascular pattern. It can be classified by the ratio of adipose and vascular tissue composition as predominantly lipomatous or angiomatous type [9]. A search of the current medical literature revealed only 15 cases of angiolipoma in the gastrointestinal tract and only 5 in the small intestine. The clinical pictures vary from asymptomatic cases to intestinal obstruction and bleeding. Surgical excision is the treatment of choice. The recurrence rate is high in cases that are inadequately resected. When the tumor can be removed completely, the prognosis is excellent [10,11].

The diagnosis of intussusception in our patient was suggested by clinical features, abdominal X-ray and CT examination. After segmental resection of ileum, we found five intraluminal masses. The histopathological findings of these masses revealed both an angiolipoma and lipomas as rare causes of intussusception.

## Conclusion

Angiolipoma and lipoma are rare benign neoplasms of gastrointestinal tract, most of them are asymptomatic and found incidentally; but may produce symptoms such as obstruction and bleeding. Surgical resection remains the treatment of choice and produces an excellent prognosis.

## Abbreviation

CT scan: computed tomography scan
